# Supplementary material for: Epsins Negatively Regulate Aortic Endothelial Cell Function by Augmenting Inflammatory Signaling
Source: Cells. 2021 Jul 29;10(8):1918. doi: 10.3390/cells10081918 (PMC8391889; doi:10.3390/cells10081918)
Supplement: Supplementary file 1 [file cells-10-01918-s001.zip › cells-1312880-supplementary.pdf]

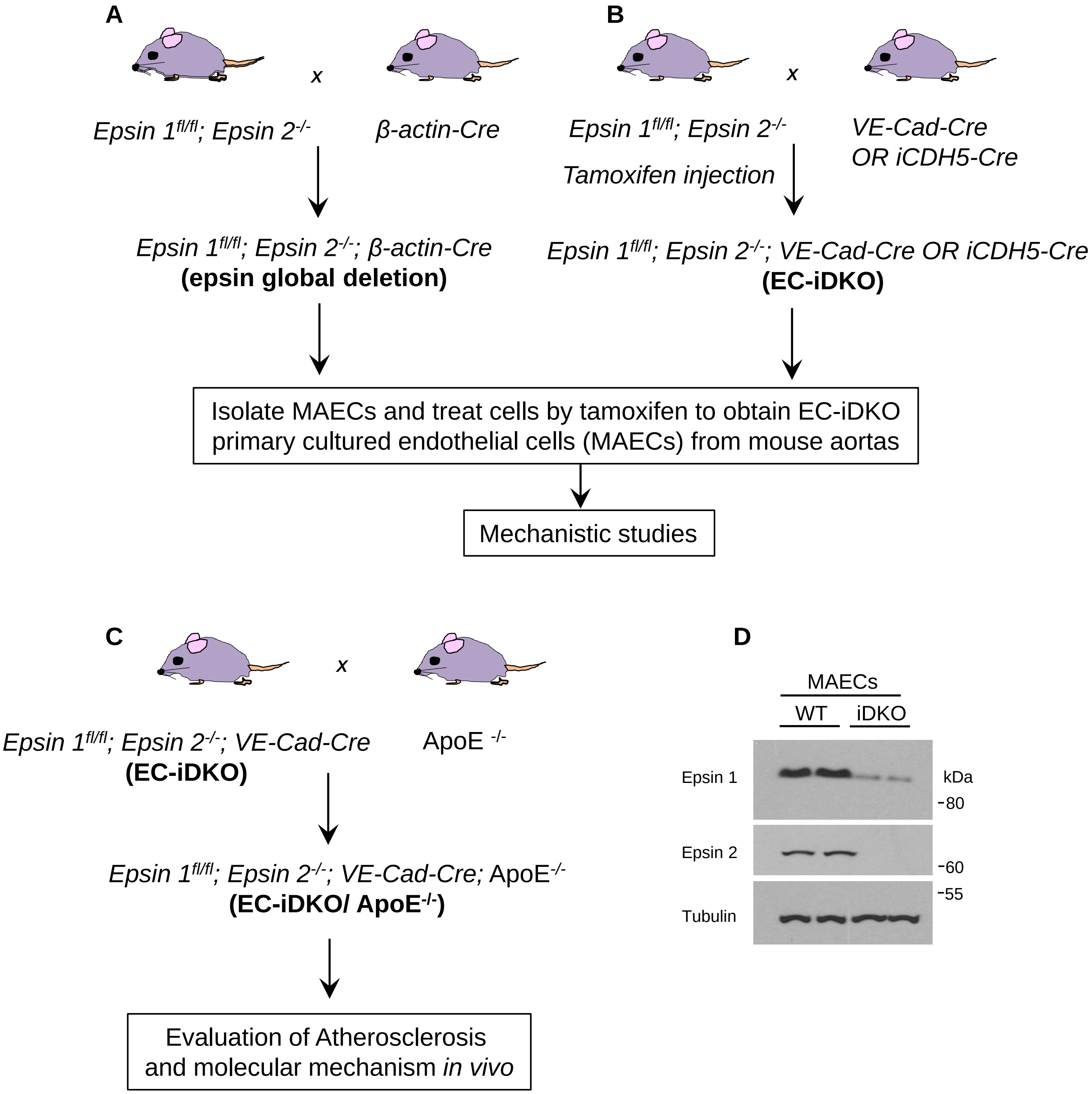

***Figure S1*. Procedures to generate primary cultured mouse endothelial cells (MAECs) from aortas, and epsin mutant mice in the ApoE-null murine atherosclerosis model.**

(A) Global epsin double knock-out (DKO) under constitutive β-actin driven Cre. (B) Conditional DKO of epsins in endothelium was driven by the vascular endothelial (VE)-cadherin promoter. (C) Generation of epsin DKO mice in the ApoE-null mouse model. (D) Creation of epsin DKO MAECs in culture. Isolated cells were treated with 5 μM tamoxifen for 4-5 days, followed by western blot analysis.

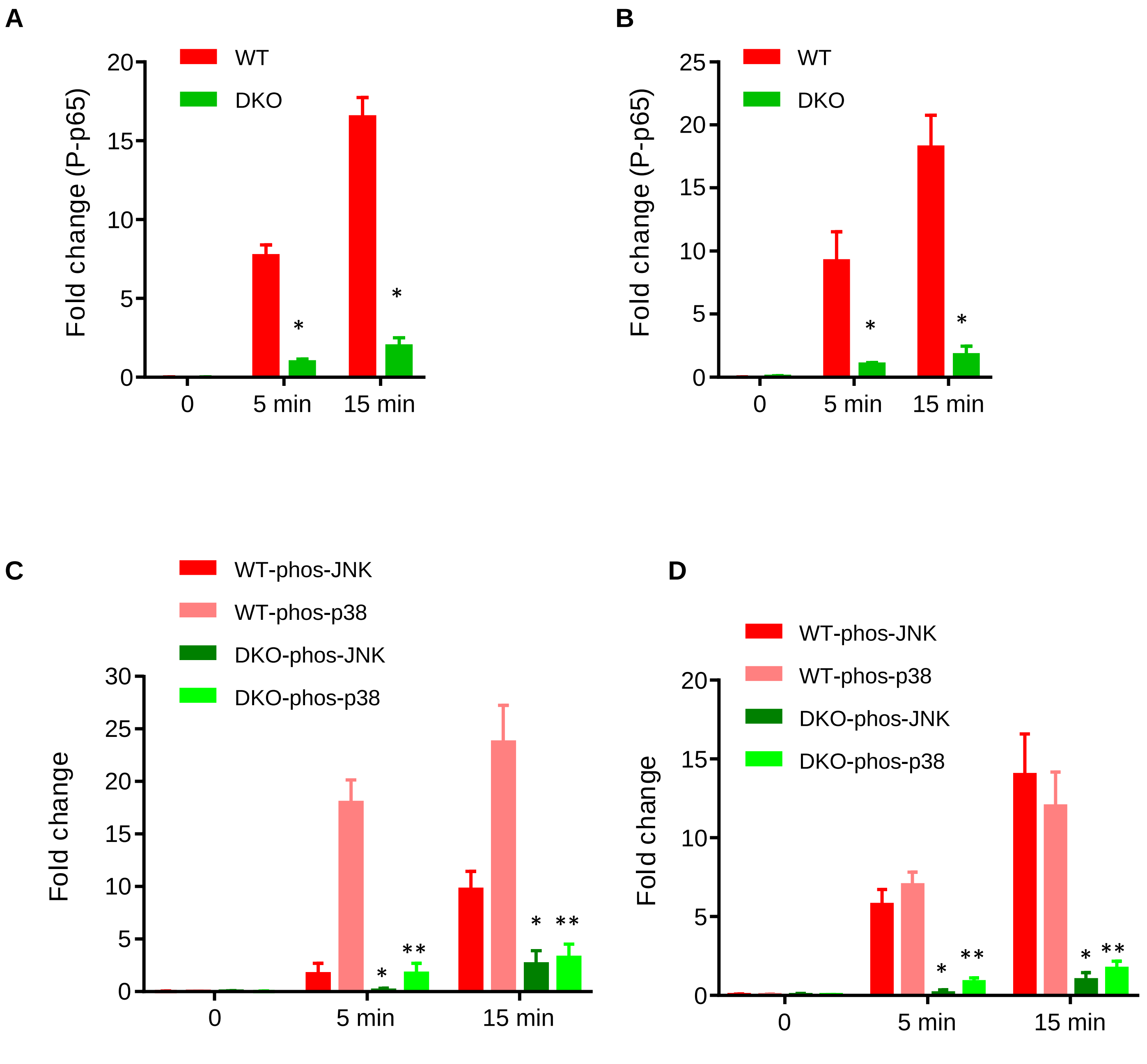

Figure S2. Quantification for Figure 1A to 1D.

(A) TNFα, phospho-p65; (B) LPS, phospho-p65; (C) TNFα, phospho-JNK and phospho-p38; (D) LPS, phospho-JNK and phospho-p38.

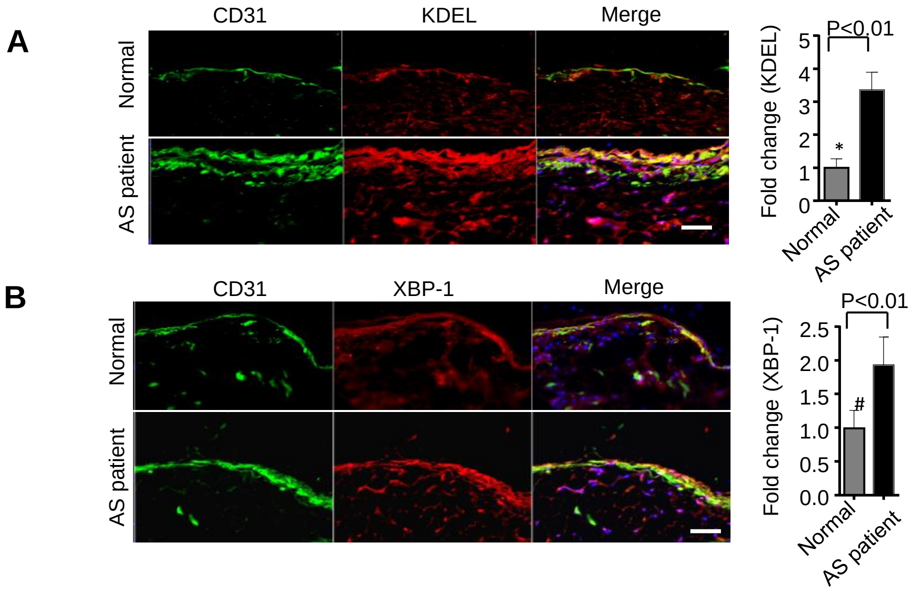

Figure S3. ER stress markers are upregulated in the aortic arches of human atherosclerosis patients, and loss of epsins in ECs attenuates ER stress.

(A, B) ER stress marker KDEL (A) and XBP-1 (B) are upregulated in atherosclerotic (AS) patients versus control patients by immunofluorescence staining (n=4-5 in each group). Scale bar: 100 μm.

A

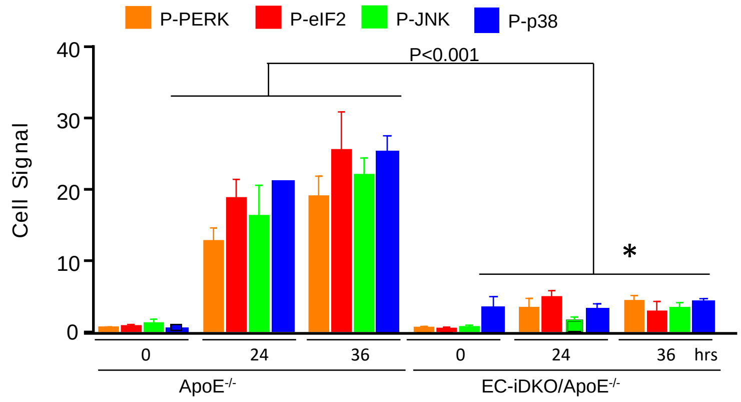

B

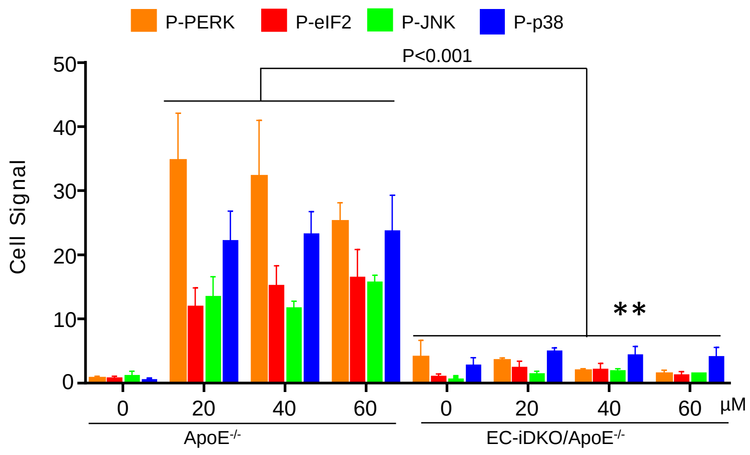

Figure S4. Quantification for Figure 2E and 2F.

MAECs were isolated from $ApoE^{-/-}$ or $DKO/ApoE^{-/-}$ mice aortas. Cells were treated with 7-KC in indicated time points (7-KC at 50 μM) or concentrations at 0, 20, 40 and 60 μM for 36 hours. Statistical analysis and quantification was shown in (A) and (B) respectively (n=4). *P* values are indicated in the histograms.

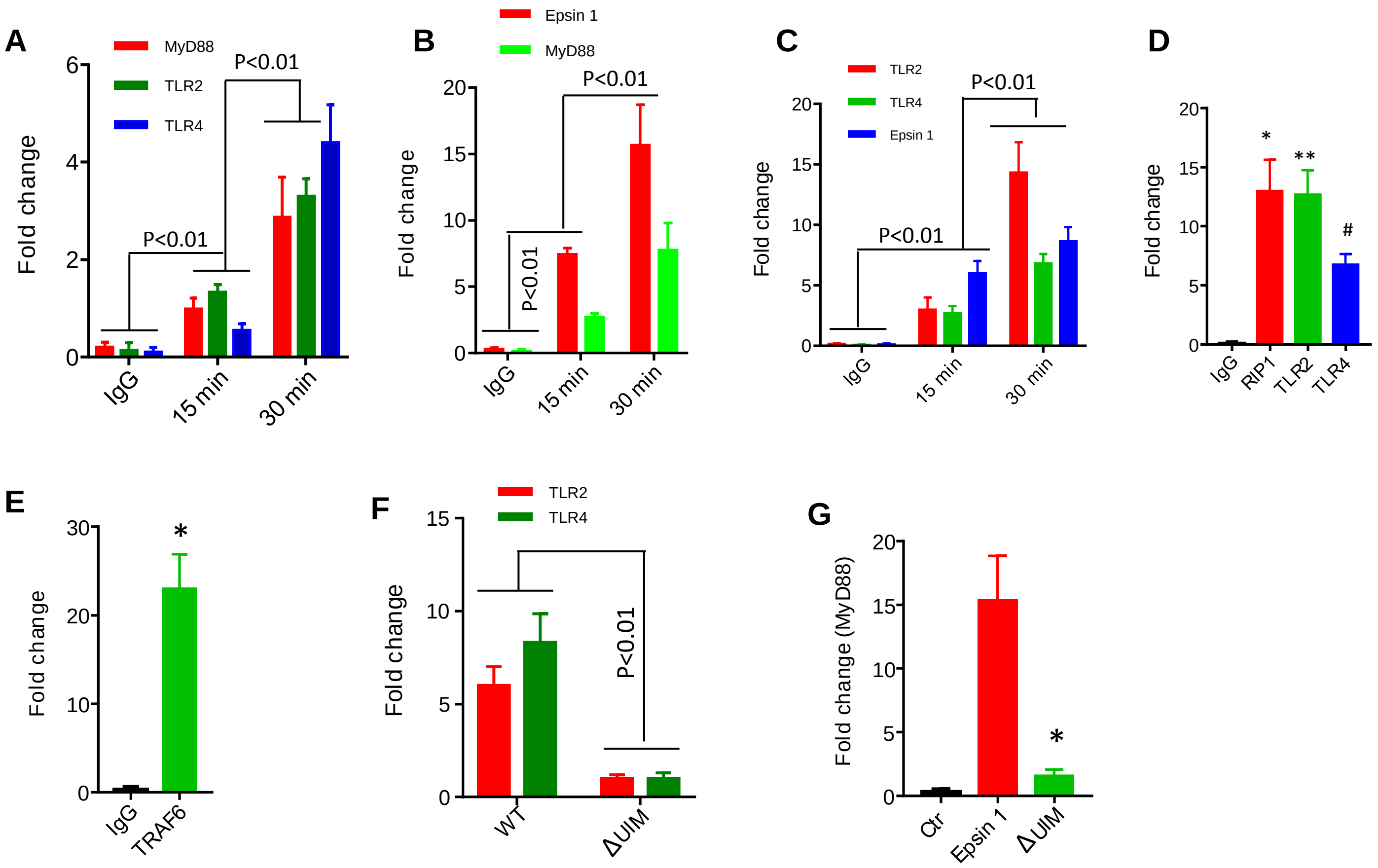

Figure S5. Quantification for Figure 6A to 6G.

(**A**) Wild type MAECs were treated with 100 ng/ml LPS for 15 or 30 min, cells were lysed for immunoprecipitation (IP) with epsin 1 antibody, and blotted with MyD88 or TLR2/4 antibodies. n=5. P<0.01; MyD88, TLR2 and TLR4 were quantified. **(B)** Treatment as in (**A**), while IP with TLR4 antibody, and blotted with Epsin 1, MyD88 and TLR4 antibodies. n=5. P<0.01; Epsin 1 and MyD88 were quantified. **(C)** Treatment as in (**A**), while IP with MyD88 antibody, and blotted with Epsin 1 and TLR2/4 antibodies. n=5. P<0.01; TLR2, TLR4 and Epsin 1 were quantified. (**D**) MAECs were treated with 100 ng/ml LPS for 30 min, IP with Epsin 1 antibody and blotted with RIP1 or TLR2/4 antibody. n=5. *, **, # P<0.01; RIP1, TLR2 and TLR4 were quantified. (**E**) MAECs were treated with LPS for 30 min, IP with Epsin 1 antibody and blotted with TRAF6. n=5. * P<0.001; TRAF6 was quantified. (**F**) Epsin 1^{wt} (HA tag) or epsin $1^{\Delta UIM}$ (HA tag) were transfected to wt MAECs by electroporation separately. After 30 hours, IP with HA antibody and blotted with HA or TLR2/4 antibody. n=3. WT vs. epsin $1^{\Delta UIM}$; TLR2 and TLR4 were quantified. **(G)** Epsin 1^{wt} (HA tag) or epsin $1^{\Delta UIM}$ (HA tag) were co-transfected to MAECs with MyD88 (Flag tag) by electroporation separately. After 30 hours, cells were lysed and IP with HA, followed by blotting with Flag or HA antibody.* P<0.001, Epsin 1 *vs*. epsin $1^{\Delta UIM}$.

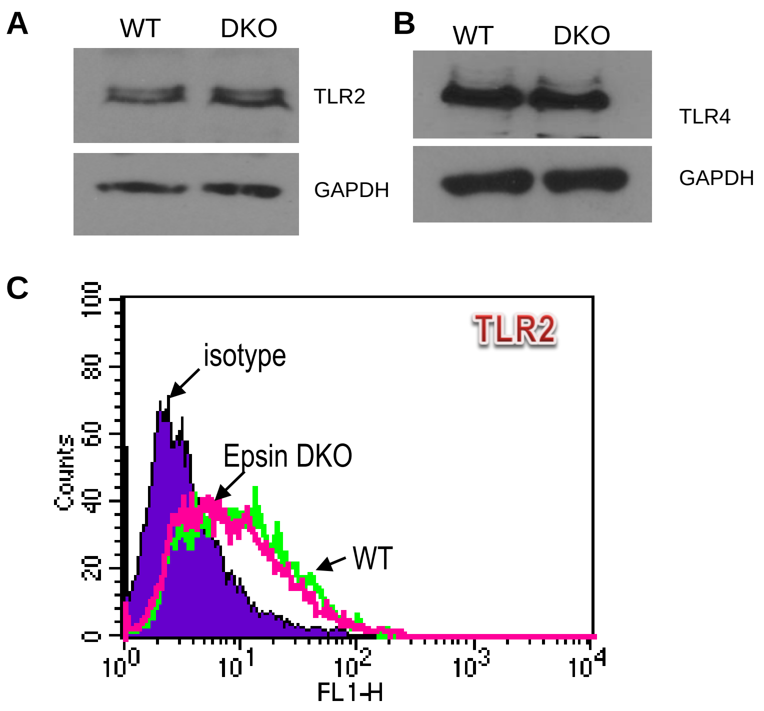

Figure S6. Loss of epsins in the endothelium did not affect the expression of TLR2 or TLR4.

(A, B) TLR2 or TLR4 expression in the MAECs of WT and DKO (n=5). (C) TLR2 is not affected by epsin loss in MAECs using flow cytometric analyses (n=5).

Table S1: qPCR primers used in this study.

Mouse Gene	Primer sequence
**ICAM-1**	Forward: GTG GTG AAG TCT GT CAA ACA GGA G<br>Reverse: CCT CCT GAG CCT TCT GTA ACT TGT
**VCAM-1**	Forward: CTC TCC CAG GAA TAC AAC GAT CTC<br>Reverse: GAC TCC AGA GTC TTC CAT CCT CAT
**P-selectin**	Forward: ATC CAG GAA GCT CTG ACG TAC TTG<br>Reverse: CAG CGT TAG TGA AGA CTC CGT ATG
**E-selectin**	Forward: GTT TGA CTG TGT GGA AGG GTA CAG<br>Reverse: GCT CAC AGG TGA AGT TAC AGG ATG
**MCP-1**	Forward: CCA CTC ACC TGC TGC TAC TCA<br>Reverse: TGG TGA TCC TCT TGT AGC TCT CC
**IFN-γ**	Forward: AAG TGG CAT AGA TGT GGA AGA AA<br>Reverse: TTG ACC TCA AAC TTG GCA ATA CT
**TNFα**	Forward: ACG GCA TGG ATC TCA AAG AC<br>Reverse: AGA TAG CAA ATC GGC TGA CG
**IL-6**	Forward: CTG GAG TAC CAT AGC TAC CTG GA<br>Reverse: CTT AGC CAC TCC TTC TGT GAC TC
**IL-1β**	Forward: TAC AAG GAG AAC CAA GCA ACG A<br>Reverse: GCT TGT GAG GTG CTG ATG TAC C
**IL-10**	Forward: GCC ACA TGC TCC TAG AGC TG<br>Reverse: CAG CTG GTC CTT TGT TTG AAA
**GAPDH**	Forward: CTC ATG ACC ACA GTC CAT GC<br>Reverse: CAC ATT GGG GGT AGG AAC AC
**β-actin**	Forward: GAT CAA GAT CAT TGC TCC TCC TG<br>Reverse: AGG GTG TAA AAC GCA GCT CA

Table S2: Body weight, blood glucose and lipid profiles of ApoE$^{-/-}$ and EC-iDKO/ApoE$^{-/-}$ mice

**Items**	**ApoE$^{-/-}$**	**EC-iDKO/ApoE$^{-/-}$**	**P value (t test)**
Body weight (g)	27.4±2.12	24.8±1.13	0.4123
Glucose (mg/dL)	127±6.90	128±14.8	0.6119
Cholesterol (mg/dL)	684.8±59.5	662±73.10	0.9419
Triglyceride (mM)	1.367±0.49	1.24±0.88	0.7463

*** n=8-10 in each group
